# Paravertebral Burkitt's Lymphoma in a Child: An Unusual Presentation

**DOI:** 10.1155/2012/891714

**Published:** 2012-11-24

**Authors:** C. Hoyoux, P. Forget, C. Piette, M. F. Dresse, B. Florkin, L. Rausin, A. Thiry

**Affiliations:** ^1^Pediatric Hematology-Oncology, CHR Citadelle, Boulevard 12ème de ligne, 4000 Liege, Belgium; ^2^Pediatric Radiology, CHR Citadelle, Boulevard 12ème de Ligne, 4000 Liege, Belgium; ^3^Pathology, CHR Citadelle, ULg, Boulevard 12ème de Ligne, 4000 Liege, Belgium

## Abstract

Paravertebral malignant tumors constitute 4.8% of cancer cases in pediatric oncology and are mostly composed of neuroblastoma (46.4%) and soft tissue sarcomas (35.7%). We describe the case of a Caucasian 6-year-old boy who was admitted for middle back pain radiated to limbs and progressively increasing weakness of the legs, suggesting a spinal cord disease. The exploration revealed two paravertebral masses extending through the neural foraminae into the epidural space. The association with elevated serum neuron specific enolase suggested at first the diagnosis of neuroblastoma, but the pathological examination revealed a Burkitt's lymphoma. This is a rare location of sporadic Burkitt's lymphoma with neurologic syndrome as first symptoms.

## 1. Introduction

 Paravertebral malignant tumors constitute 4.8% of cancer cases in pediatric oncology and are mostly composed of neuroblastoma (46.4%) and soft tissue sarcomas (35.7%) [1]. 

 Neuroblastoma (NBL), ganglioneuroblastoma, and ganglioneuroma are embryonal tumors of the sympathetic nervous system which derive from the primitive neural crest. Those primary tumors can arise at any localization coinciding with normal sympathetic nervous system structure, for example, the adrenals, the sympathetic chain, or abdominal paraganglia. Thoracic, abdominal, and pelvic paravertebral tumors can extend through the neural foraminae and compress nerve roots and the spinal cord. Catecholamines are usually elevated in the urine and serum neuron-specific enolase is a useful but not specific marker.

 Soft tissue sarcomas (STSs) of the paravertebral region represent about 3.3% of the pediatric STS. These lesions tend to invade the spinal extradural space and are composed of extraosseous Ewing's sarcomas or undifferentiated sarcomas in 55% of the cases [2].

Childhood non-Hodgkin lymphomas (NHLs) differ from the common types of adult lymphoma by their pathological, immunological, and clinical characteristics. While adult lymphomas are more commonly of low or intermediate grade, more than 90% of childhood NHL are of high grade and belong to four major histological subtypes: Burkitt's lymphoma (BL), diffuse large B-cell lymphoma, lymphoblastic lymphoma, and anaplastic large cell lymphoma. Two types of BL can be defined: the sporadic (Europe and the Americas) and the endemic (equatorial Africa) varieties. The sporadic form typically occurs in the abdomen—most often the ileo-caecal area—that is, affected in more than 90% of the children with sporadic BL. Other sites include cervical lymphadenopathies, testis, bone, skin, bone marrow and central nervous system. Paravertebral involvement is a very rare presentation (<2%) of BL [3]. 

## 2. Case Presentation

**Figure 1:**
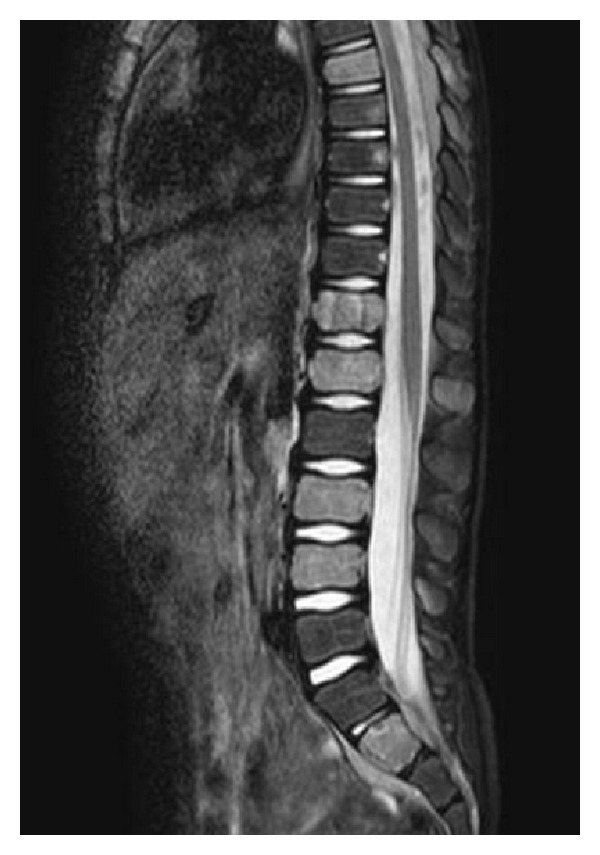
Spine MRI (median sagittal STIR): heterogeneous captation in some vertebrae (T7, T12, L1, L3, L4, S2), suggesting bone marrow infiltration.

**Figure 2:**
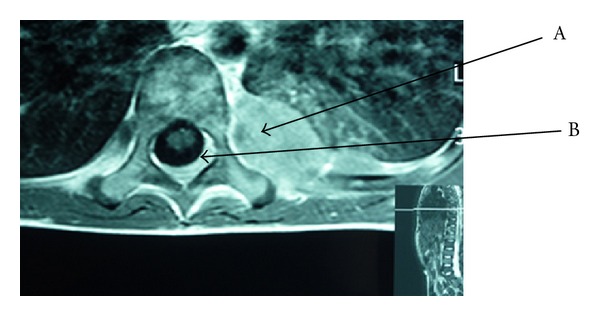
MRI (axial T1): dorsal paravertebral mass (T7) (A) with extra dural deposits and compressin of the thecal sac (B) from tumor spread.

**Figure 3:**
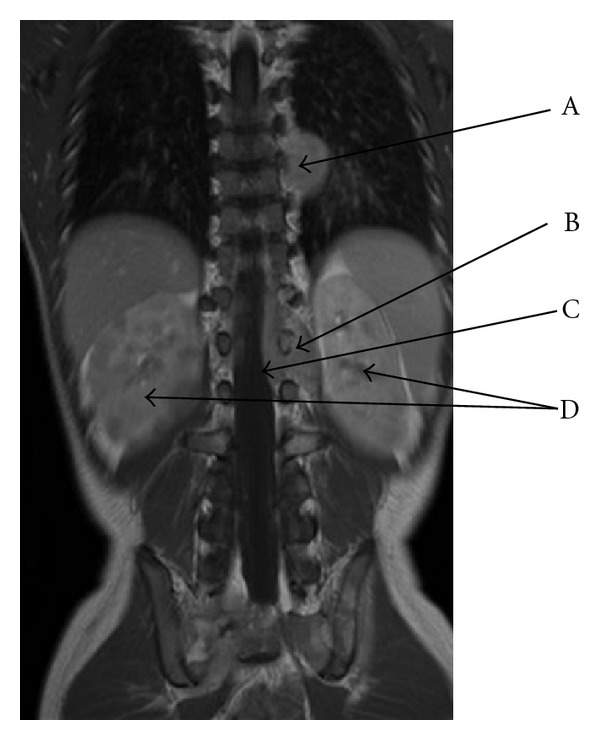
MRI (coronal T1 gadolinium): left dorsal paravertebral tumor (T7–T9) (A) and left dorso-lumbar paravertebral mass (B) with epidural compression (C) through infiltration via intervertebral foraminae (T12–L2). Bilateral nodular nephromegaly (D).

**Figure 4:**
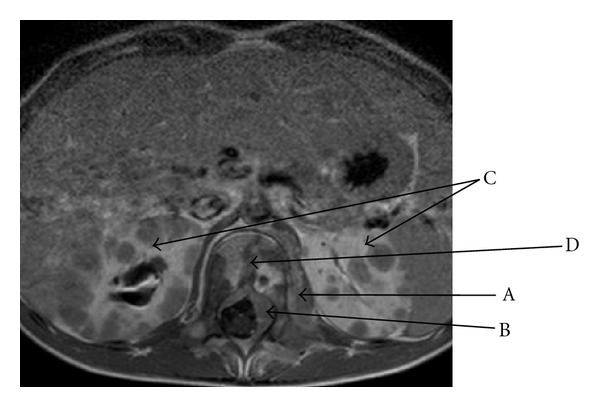
MRI (axial T1 gadolinium) lumbar paravertebral tumor (A) with epidural compression (B) through foraminae infiltration; bilateral nodular nephromegaly (C); heterogeneity captation of the vertebra (D), suggesting bone marrow infiltration.

A European 6-year-old male was admitted in our hospital for nonspecific constant middle back pain radiated to the limbs. Complaints include discomfort, progressive weakness of legs with abnormal sensations leading to unusual walk and limping. Symptoms increase with bending back and forward. He also suffered from chills, fever, anorecia and weight loss for 6 weeks without any infectious or traumatic context anorexia. On examination he was pale, irritable, in poor condition and painful. No tingling was described; no bladder or bowel loss of control was present. Cutaneous sensitivity and motor reflexes seemed normal. Abdominal palpation was normal. Blood investigations showed isolated anemia, major systemic inflammatory response (ESR = 109), and elevation of LDH (2328 U/L) and neuron-specific enolase (NSE = 69.5 ng/mL). Alkaline phosphatase and BUN were normal. Total spine X-ray and CT scan were normal and excluded any osseous vertebral abnormality; bone 99 Tc-scintigraphy was also normal. Sagittal spine MRI showed heterogeneity of gadolinium captation in some vertebrae, suggesting bone marrow infiltration (Figure 1). Ultrasonography and MRI showed two paravertebral tumours: an extrapleural left homogenous paravertebral mass located along dorsal vertebrae T7–T9, measuring 52 × 22 mm and another paravertebral mass located in dorso- lombar (T 12–L2) measuring 40 × 10 mm. Those two tumours extended through the neural foraminae into the epidural space (Figures 2, 3, 4). The cerebrospinal fluid (CSF) analysis revealed the absence of tumoral cells and both proteinorachy and CSF NSE were normal. Extension assessment showed nodular bilateral nephromegaly (Figures 3 and 4) without renal dysfunction.

The association of paravertebral masses, serum NSE elevation and probable bone marrow infiltration suggested at first a neuroblastoma diagnosis.

**Figure 5:**
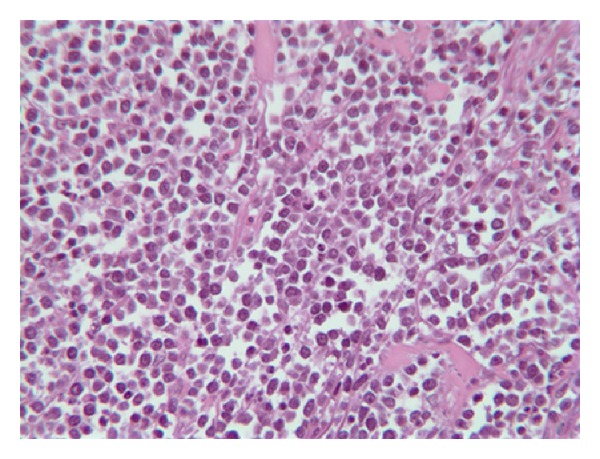
Pathology of tumour specimen instead of biopsy of the tumour anatomopathology (HE—400x): intermediate size homogenous basophilic cells with hyperchromatic nuclei containing nucleoli: Burkitt's lymphoma.

A transparietal tumoral biopsy was performed and pathological examinations revealed homogenous basophilic cells of intermediate size with hyperchromatic nuclei containing nucleoli (Figure 5). The tumor cells had a high mitotic activity and apoptotic debris were noted. Immunophenotypic features demonstrated mature B cells positive for CD45, CD20, CD10 and negative for TdT and CD99. Lymphoma cells were monotypically positive IgM + D + Lambda, BCL2 negative, and BCL6 positive. Translocation 8;14 was identified, confirming the definitive diagnosis. The EBV RNA was not found in the specimen.

The patient was immediately treated by chemotherapy alone. Neurological symptoms rapidly resolved and the patient is now well and in first complete remission at 5 years.

## 3. Discussion

In our patient, neurological symptoms were the first manifestations of a primary paravertebral tumor. The initial presentation with back pain, progressive weakness of legs, and difficulties to walk suggested a spinal cord disease (SCD). Plain X-ray, spine CT scan, and bone scintigraphy were normal, excluding a lesion originating from the vertebra. CSF was also normal, without pleocytosis or hyperproteinorachy. MRI demonstrated two paravertebral masses extending through the neural foraminae and compressing the spinal nerve roots. 

The differential diagnosis of paravertebral masses includes mainly infectious and tumoral processes. Paravertebral abscesses usually develop after untreated spine infections involving vertebral body and adjacent disk interspace; this diagnosis was excluded in our patient. A variety of tumors should be differentiated when paraspinal mass is discovered, including neurogenic, neuroendocrine tumors, menigiomas, ependymomas, sarcomas, processes arising from lymphoid, connective, and osseous tissues. In children, the most frequent are sympathetic nervous system tumors (neuroblastomas), extraskeletal form of Ewing sarcomas which show a predilection for the paravertebral and costal niches and peripheral nerve sheath tumors, occurring usually in older patients. 

The association of paravertebral masses and serum NSE elevation in our child suggested at first the diagnosis of neuroblastoma. 

 Neuron specific enolase (NSE) is an isozyme of the glycolytic pathway specifically found in brain and neuroendocrine tissue. It is a tumoral marker for neuroendocrine neoplasms, namely, neuroblastoma in pediatric oncology. Nevertheless, the presence of NSE has also been demonstrated in miscellaneous tumors, including malignant lymphomas [6, 7] and seems to be a sensitive marker in the early diagnosis of brain injuries [8]. In our patient, NSE was elevated in serum but normal in CSF. 

 CNS extension occurs in 8.8% of childhood BL and most often consists of meningeal disease [9]. Spinal localization of BL usually results from primary spinal epidural tumor, as described by others [4, 10–14]. In those cases, pleocytosis and proteinorachy are elevated in CSF and vertebral are frequently involved and sometimes collapsed. In our patient, CNS extension and SCD resulted from the epidural space infiltration through the intervertebral foraminae, leading to the compression of the thecal sac, without any tumoral CSF involvement. Paravertebral lymphomas are rarely reported and frequently misdiagnosed [5, 15].

Spinal cord disease is usually diagnosed at a late stage. Early symptoms may fluctuate in intensity and the findings on neurological examination may even be normal in the presence of a spinal tumoral mass. Clinical features of SCD include back pain, motor deficits, sensitivity abnormalities, sphincter disturbances, and later on paraplegia [11–14]. As BL is a rapidly growing tumor, early diagnosis is sometimes difficult, resulting in permanent neurological deficits.

 Bilateral nodular nephromegaly—as seen in our patient—has been described in Burkitt's lymphoma [16]. 

## 4. Conclusions

Paravertebral malignant tumors constitute 4.8% of cancer cases in pediatric oncology and are mostly composed of neuroblastoma (46.4%) and soft tissue sarcomas (35.7%) [1]. Paraspinal involvement is a very rare presentation (<2%) of BL. 

 Even though malignancies involving the spine are not common, they account for a disproportionate degree of morbidity. Remission may be achieved, but neglected neurological deficits almost always result in permanent disability. Since the outcome of SCD is primarily determined by the patient's neurologic status at treatment initiation, the goal must be to establish the underlying diagnosis before irreversible spinal cord damage. Owing to the effectiveness of chemotherapy in BL, surgery should be rarely considered and should be reserved to rapid neurological deterioration or to diagnostic purposes. 

 Despite this unusual location, BL should be included in the differential diagnosis in patients with nerve root symptoms and in paravertebral processes.
